# Editorial: The bodily self and its alterations: psychopathological, neural and theoretical aspects

**DOI:** 10.3389/fpsyg.2024.1447443

**Published:** 2024-07-01

**Authors:** Francesca Ferroni, Daniela Rabellino, Claudia Mazzeschi, Martina Ardizzi

**Affiliations:** ^1^Department of Medicine and Surgery, Unit of Neuroscience, University of Parma, Parma, Italy; ^2^Department of Psychiatry, Western University, London, ON, Canada; ^3^Master of Arts in Counselling Psychology, Yorkville University, Fredericton, NB, Canada; ^4^Department of Philosophy, Social Sciences, Humanities and Education, University of Perugia, Perugia, Italy

**Keywords:** anorexia nervosa, multisensory integration, obesity, schizotypy, self-disturbances

We experience our body in movement, interacting with the world. Thus, our awareness of the body is intimately tied to movement and interaction with the environment. Movement is, indeed, the glue that binds the body and the self together as it is closely connected to the brain's creation of a sense of bodily self (Gallese and Sinigaglia, 2010, 2011a,b). The self is constructed through the processing of multisensory and motor signals, which generate a sense of ownership and agency. Consequently, the motor integration of multisensory bodily inputs underpins the non-conceptual and pre-reflective core of the self, known as the bodily self.

Actions and body are also the main ways humans express their social inclinations, forming early social cognition (Von Hofsten, 2007). A coherent bodily self is, indeed, essential for proper interaction with the environment and other people, thus supporting the concept of an inherently social bodily self (Ferroni and Gallese, 2022). This is particularly evident in clinical populations where the bodily self and its underlying mechanisms are disrupted (e.g., Eshkevari et al., 2014; Keizer et al., 2016; Ardizzi et al., 2020; Rabellino et al., 2020; Ferroni et al., 2022; Gallese et al., 2024). However, bodily self-alterations appear to be unspecific, despite the phenotypic differences between these disorders. Indeed, although there has been a growing interest in the study of the bodily self and the processes beyond it over the past 30 years, many questions are still unanswered.

The present Research Topic investigates the link between sensory perception, multisensory integration, and bodily self-representation, shedding new light on the diverse panorama of alterations affecting bodily self across different clinical populations. Below is a brief overview of original research contributions highlighting the significance of a coherent bodily self for healthy psychological and social functioning and illustrating the consequences of its disruption.

Torregrossa et al. examined multidimensional schizotypy and discovered that individuals with higher negative schizotypy experience more ambiguous and less coherent emotional sensations in their bodies. This underscores the importance of further investigating these embodied emotional patterns to better understand their impact on the functionality and quality of life of individuals with schizotypy and schizophrenia. This study also underscores the need to adopt a continuous view of psychopathology and its underlying processes.

Alterations in body image, self-recognition, and bodily self-perceptions (Keizer et al., 2013; Crucianelli et al., 2016; Campione et al., 2017; Davidovic et al., 2018) have been observed in anorexia nervosa, a heavily frequent and notably precocious clinical condition. Ambrosecchia et al. investigated bodily self-recognition in individuals with restrictive anorexia nervosa. The authors found a preserved implicit self-advantage in anorexia patients, despite the weaker processes associated with motor imagery of body parts compared to those found in healthy controls. Moreover, they found that the bodily self, at an explicit level, together with the integration and distinction between self and other, is altered in anorexia, shedding light on how damage to one of the self-layers may lead to different symptomatology and opening new therapeutic approaches for bodily self-disorders.

Tagini et al. investigated the perception of affective touch in another disordered eating behavior, namely, obesity. Affective touch, which refers to gentle, caressing touch that activates C-tactile afferents, plays a crucial role in forming and maintaining the bodily self by enhancing the integration of multisensory information (Crucianelli et al., 2013). In this Research Topic, Tagini et al. found that women with obesity reported fewer satisfying affective touch experiences during their formative years, although their current perception of such touch remains unaffected. This early deprivation may contribute to altered emotional and social interactions, impacting their bodily self-perception.

Bodily self is still a crucial issue for functional neurologic disorders (FNDs), which are conditions in which patients experience neurological symptoms that cannot be explained by medical or neurological disease. These symptoms are real and can cause significant distress and impairment, but they arise from functional issues in the nervous system rather than structural damage or disease. Onofrj et al. highlight that neurologists may feel inadequately equipped to handle FNDs due to a lack of resources and specific training. For this reason, the authors call for better education and resources for neurologists to improve patient outcomes and effectively address the unique needs of FND patients. In this light, a coherent bodily self is crucial for overall psychological and social functioning, which is potentially compromised in FND patients. Future research should delve into this aspect and explore potential impairments in other layers of self in these patients to provide clinicians with clinical evaluation and tests related to bodily self-damage.

In the healthcare field, Laricchiuta et al. highlight the relevance of mind/body interventions that focus on the multisensory and motor integration processes underlying the bodily self in addressing the multifarious negative consequences of traumatic experiences that crystallize over time and can severely disrupt the bodily self, resulting in difficulties in environmental interaction.

The studies on the present Research Topic collectively emphasize the crucial role of the bodily self in shaping our interactions and highlight how its disruption can lead to psychological and social impairments. All these different disorders underscore the diverse ways in which the bodily self can be compromised, providing insights into early social cognition and emphasizing the necessity for targeted therapeutic interventions. Future research should continue exploring the interplay between multisensory integration, motor processes, and the bodily self to develop effective strategies for improving it in clinical populations.

## Author contributions

FF: Conceptualization, Writing—original draft, Writing—review & editing, Supervision. DR: Conceptualization, Writing—review & editing. CM: Conceptualization, Writing—review & editing. MA: Conceptualization, Writing—review & editing, Supervision.
